# The Role of Bromodomain and Extraterminal (BET) Proteins in Controlling the Phagocytic Activity of Microglia In Vitro: Relevance to Alzheimer’s Disease

**DOI:** 10.3390/ijms24010013

**Published:** 2022-12-20

**Authors:** Marta Matuszewska, Magdalena Cieślik, Anna Wilkaniec, Marcin Strawski, Grzegorz A. Czapski

**Affiliations:** 1 Department of Cellular Signalling, Mossakowski Medical Research Institute, Polish Academy of Sciences, ul. Pawińskiego 5, 02-106 Warsaw, Poland; 2 Laboratory of Electrochemistry, Faculty of Chemistry, University of Warsaw, ul. Pasteura 1, 02-093 Warsaw, Poland

**Keywords:** BET, microglia, BV2, phagocytosis, Alzheimer’s disease

## Abstract

The correct phagocytic activity of microglia is a prerequisite for maintaining homeostasis in the brain. In the analysis of mechanisms regulating microglial phagocytosis, we focused on the bromodomain and extraterminal domain (BET) proteins: Brd2, Brd3, and Brd4, the acetylation code readers that control gene expression in cooperation with transcription factors. We used pharmacological (JQ1) and genetic (siRNA) inhibition of BET proteins in murine microglial cell line BV2. Inhibition of BET proteins reduced the phagocytic activity of BV2, as determined by using a fluorescent microspheres-based assay and fluorescently labelled amyloid-beta peptides. Gene silencing experiments demonstrated that all brain-existing BET isoforms control phagocytosis in microglia. From a set of 84 phagocytosis-related genes, we have found the attenuation of the expression of 14: *Siglec1*, *Sirpb1a*, *Cd36*, *Clec7a*, *Itgam*, *Tlr3*, *Fcgr1*, *Cd14*, *Marco*, *Pld1*, *Fcgr2b*, *Anxa1*, *Tnf*, *Nod1,* upon BET inhibition. Further analysis of the mRNA level of other phagocytosis-related genes which were involved in the pathomechanism of Alzheimer’s disease demonstrated that JQ1 significantly reduced the expression of *Cd33*, *Trem2*, and *Zyx*. Our results indicate the important role of BET proteins in controlling microglial phagocytosis; therefore, targeting BET may be the efficient method of modulating microglial activity.

## 1. Introduction

Microglial cells, which represent the immune system in the central nervous system (CNS), play a crucial role in maintaining tissue homeostasis, a prerequisite of a healthy brain [1]. As resident brain macrophages, they are equipped with a wide array of receptors and constantly examine the local microenvironment, searching for signals indicating disorder, like infection or cell death. Detection of any sign of imbalance triggers a respective microglial reaction. However, microglia also have important functions in a healthy brain. During proper brain development, microglia shape neuronal connections by phagocyting redundant synapses, unnecessary axons, dendrites, and entire neurons [2,3,4,5,6,7,8]. Self-renewal and homeostasis of microglia are maintained by several factors, like colony stimulating factor 1 (CSF1) and interleukin-34 (IL-34) [9,10]. The deregulation of those factors results in microglial dysfunction and is associated with many neurodegenerative diseases, including Alzheimer’s disease (AD), a form of dementia that usually appears in the elderly, characterized by partial or complete loss of memory and cognition [11,12]. Recently, it was postulated that excessive phagocytosis by microglial cells activated by disturbed neuronal signalling has a detrimental role in the progression of AD-related pathology [13]. Activation of microglia and microglia-mediated phagocytosis must be tightly controlled in time and space, in other cases, it may be detrimental to the tissue. Thus, identifying the molecular targets for inhibition of aberrant microglial phagocytosis could be an efficient method of attenuating inflammation. Among many targets, bromodomain and extraterminal (BET) proteins seem to be especially interesting. In the CNS, the expression of three proteins belonging to the BET family, Brd2, Brd3, and Brd4, was observed. They act as epigenetic readers of acetylation code, controlling transcription activation and elongation by the recruitment of RNA polymerase II (Pol II) and the positive transcription elongation factor (P-TEFb) [14]. They are also coactivators of p65 (RelA), a subunit of the major inflammation-related transcription factor NF-κB. BET proteins were demonstrated to be involved in the pathomechanism of malignant diseases, immunological, neurological, and cardiovascular syndromes, as well as sepsis [15,16,17,18,19]. They also directly regulate inflammatory responses [20]. BET proteins are involved in the activation of innate immunity, and their inhibition has been demonstrated to attenuate pro-inflammatory processes [15]. A pan-inhibitor of BET family JQ1 was shown both in vitro and in vivo to inhibit lipopolysaccharide (LPS)-stimulated expression of cytokines, including IL-1β, IL-6, IL-10, IL-18, tumor necrosis factor α (TNF-α), C–C motif chemokine ligand 2 (CCL2), CCL3, CCL4, and monocyte chemoattractant protein-1 (MCP-1) [21,22]. Accordingly, the inhibition of BET proteins prevents LPS-induced expression of proinflammatory cytokines in mouse bone marrow-derived macrophages [22]. Therefore, the BET family may become an important target in the treatment of inflammation-related diseases [23]. It is very important that we gain a deeper understanding of the overlapping and distinct functions of BET proteins in physiology and disease, as they may contribute to the activation of transcription of several genes, including those which regulate phagocytosis [24]. Therefore, the aim of our study was to analyse the role of BET proteins in regulating phagocytosis in microglial cells. The results demonstrated that Brd2, Brd3, and Brd4 control the transcription of phagocytosis-related genes, and BETs’ inhibition may be the efficient method of modulating microglial phagocytosis.

## 2. Results

**Figure 1:**
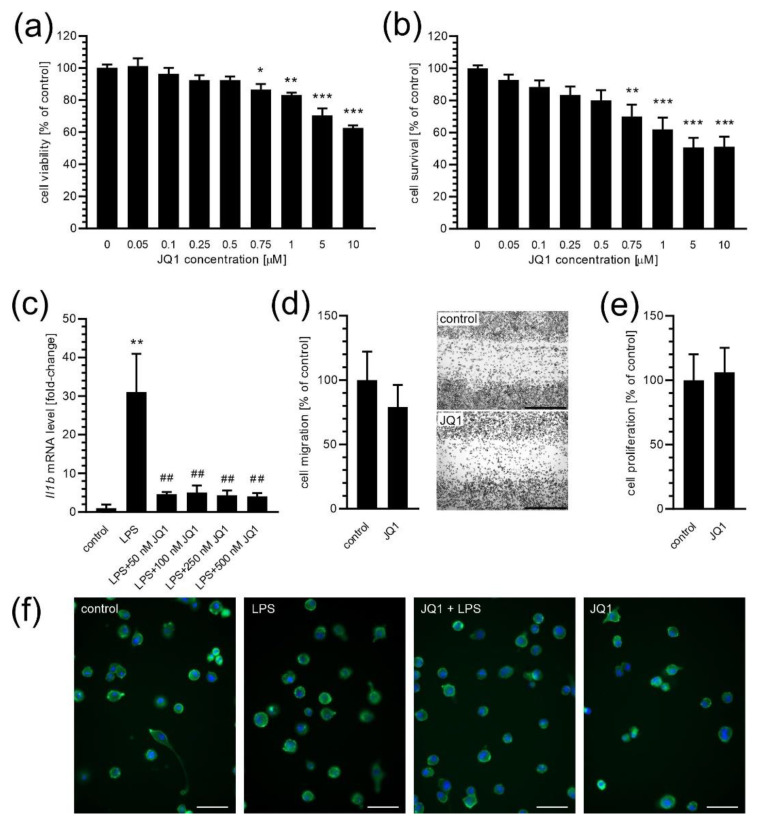
The effect of BET inhibitor JQ1 on microglial BV2 cells. (**a**) BV2 cells were incubated for 24 h in the presence of JQ1, and then an MTT assay was performed to analyse cell viability (*n* = 3–5). (**b**) BV2 cells were incubated for 24 h in the presence of JQ1, and then PI staining assay was performed to analyse cell survival (*n* = 7). (**c**) BV2 cells were incubated for 2 h in the presence of LPS (100 ng/mL) and JQ1; then, the mRNA level for *Il1b* gene was determined by using qPCR (*n* = 3–4). (**d**) BV2 cells were incubated for 24 h in the presence of 50 nM JQ1, and then a scratch assay was performed to analyse cell migration (*n* = 6). Typical images are presented. The scale bar: 1 mm. (**e**) BV2 cells were incubated for 24 h in the presence of 50 nM JQ1; then, the cell number was counted to analyse cells’ proliferation (*n* = 6–7). (**f**) The morphology of BV2 cells in control conditions and in the presence of JQ1 (50 nM, 24 h) and LPS (100 ng/mL, 2 h). Cells were stained with Hoechst 33342 and Cell Mask Green Actin Tracker. The scale bar: 50 µm. Means ± SEM are presented. *, **, *** *p* < 0.05, 0.01, and 0.001, respectively, compared to the control. ## *p* < 0.01, compared to the LPS-treated group. Statistical analysis was performed by using a one-way ANOVA followed by the Bonferroni post hoc test (**a**–**c**) or the Student *t*-test (**d**,**e**).

In our study, we used a murine BV2 cell line to determine the role of BET proteins in controlling the phagocytic function of microglia. To inhibit the activity of BET proteins, we used the pharmacological inhibition by JQ1 or a genetic (siRNA) approach. First, we examined the effect of different concentrations of BET inhibitor JQ1 on BV2 cells’ viability to establish the optimal non-toxic concentration (Figure 1). By using an MTT assay, we observed that treatment with JQ1 for 24 h with concentrations below 0.75 µM had no impact on cell viability (Figure 1a). Similar results were obtained by using a propidium iodide (PI)-staining assay, where JQ1 at concentrations higher than 0.5 µM displayed toxic properties (Figure 1b). Therefore, LPS-stimulated cells were pre-treated with JQ1 at a non-toxic range (50–500 nM) to determine the effective inhibitory concentration. As shown in Figure 1c, all tested concentrations of JQ1 efficiently reduced LPS-evoked interleukin 1β expression. On the basis of these data, 50 nM concentration of the JQ1 was chosen for further studies. At this concentration, 24 h incubation with JQ1 did not impact BV2 cells’ migration (Figure 1d), proliferation (Figure 1e), and morphology (Figure 1f). Additionally, we did not observe any change in cell morphology after 2 h of treatment with LPS (Figure 1f)—perhaps this time period is too short to observe morphological changes. We also verified the potency of other BET inhibitors, and the obtained results are presented in Supplementary Figure S1.

**Figure 2:**
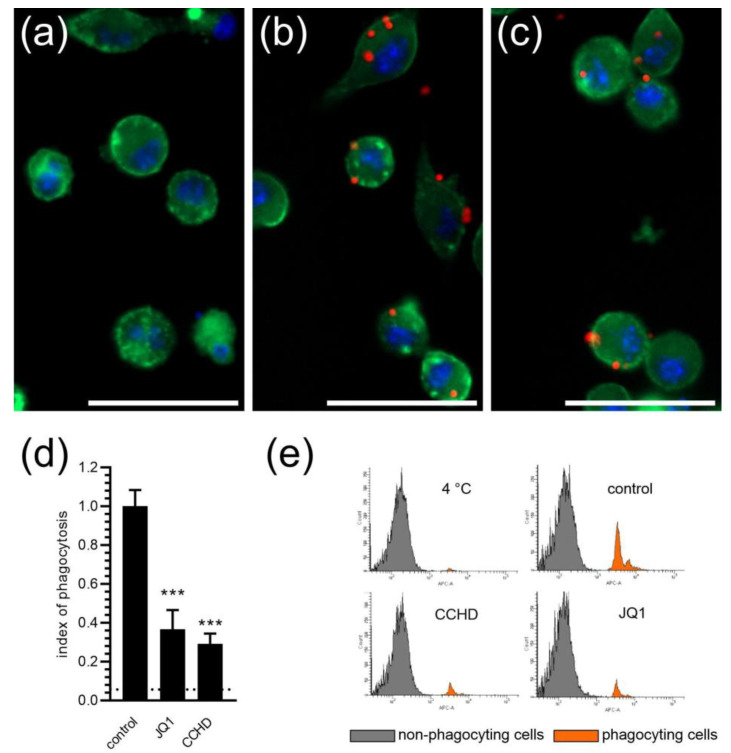
The effect of the BET inhibitor on phagocytosis of fluorescent microspheres by microglia. BV2 cells were incubated for 2 h without FMS (**a**), with FMS in the absence of JQ1 (**b**) or after 24 h of preincubation with 50 nM JQ1 (**c**). Cells are stained with green cytoplasmic dye and blue nuclear dye. Fluorescent microspheres are orange. The scale bar: 50 µm. (**d**) Flow cytometric analysis of the effect of BET inhibitor and CCHD on FMS’ phagocytosis by microglia. The level of non-specific labelling is shown with a dotted line. (**e**) Typical flow-cytometric histograms of non-phagocytic and phagocyting BV2 cells. Means ± SEM are presented. *n* = 11–13; *** *p* < 0.001, compared to the control. Statistical analysis was performed by using a one-way ANOVA followed by the Bonferroni post hoc test.

To analyse the role of BET proteins in controlling the phagocytic function of BV2 cells, we used the fluorescent microspheres (FMS)-based assay. Confocal microscopy analysis demonstrated that, in control conditions, BV2 cells efficiently ingested FMS, but 24 h pre-treatment with JQ1 appeared to decrease the amount of engulfed FMS (Figure 2a–c). For quantitative assessment, we used the established method based on the flow cytometry analysis (Figure 2d,e), and to verify the accuracy of this method, we used phagocytosis inhibitor cytochalasin D (CCHD; 2 µM) as a positive control. We observed that CCHD significantly reduced the number of microglial cells with the ingested FMS. We also established the level of the non-specific binding of FMS to cellular membranes by incubating the cells at 4 °C, the conditions in which microglial cells phagocytosis is completely inhibited [25]. As demonstrated in Figure 2d,e, a very small fraction of BV2 cells was labelled with FMS at 4 °C; therefore, we recognized the non-specific binding of FMS to cells as negligible. Our flow-cytometric analysis demonstrated that inhibition of BET proteins with 50 nM JQ1 significantly reduced the fraction of FMS-positive BV2 cells, indicating that BET proteins contribute to the regulation of microglial phagocytosis. 

**Figure 3:**
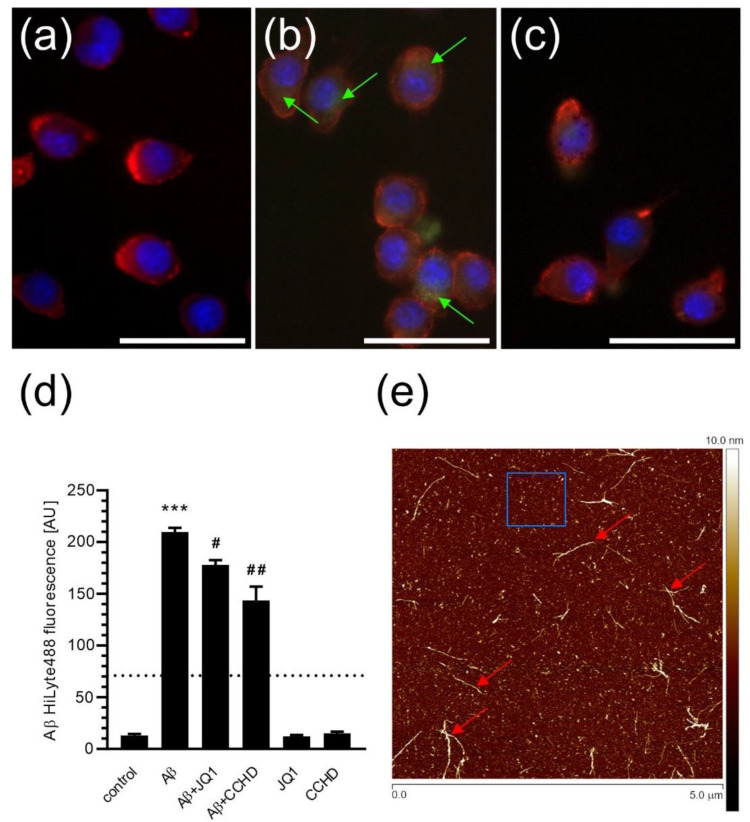
The effect of BET inhibitor JQ1 on Aβ phagocytosis by microglia. BV2 cells were preincubated with 50 nM JQ1 for 24 h or with 2 µM CCHD for 30 min, and then fluorescently labelled Aβ_1–42_ (Aβ HiLyte488; green on the pictures) was added. After 2 h incubation, cells were stained with red cytoplasmic dye and blue nuclear dye. (**a**) Control, (**b**) cells incubated with Aβ, (**c**) cells preincubated with JQ1 and incubated with Aβ. The scale bar: 50 µm. Green arrows point to intracellular Aβ HiLyte488 staining. (**d**) Flow-cytometric analysis of the effect of the BET inhibitor and CCHD on Aβ phagocytosis by microglia. The dotted line shows the level of unspecific labelling with Aβ (at 4 °C). (**e**) The typical AFM picture of Aβ after 2 h incubation at 37 °C. Example oligomers were marked with a blue rectangle; red arrows indicate representative protofibrils. Means ± SEM are presented. *n* = 6–8; *** *p* < 0.001, compared to control, #, ## *p* < 0.05 and *p* < 0.01, compared to Aβ group. Statistical analysis was performed by using a one-way ANOVA followed by the Bonferroni post hoc test.

Microglial phagocytosis was also assessed in the BV2 cells pre-treated with 50 nM JQ1 for 24 h and subsequently exposed to fluorescently labelled Aβ_1–42_ for 2 h. During 2 h of incubation with Aβ, we did not observe changes in the morphology of the cells. The Aβ species were efficiently phagocytosed by BV2 cells during 2 h of incubation, as visualized using confocal microscopy (Figure 3a–c, green arrows) and quantified using flow cytometry (Figure 3d). Preincubation with JQ1 significantly reduced the amount of Aβ ingested by microglial cells (Figure 3c,d), indicating the important role of BET proteins in controlling Aβ phagocytosis by microglia. As visualized using atomic force microscopy (AFM), 2 h of incubation of Aβ_1–42_ in a cell culture medium at 37 °C resulted in the formation of a mixture of low-molecular-weight prefibrillar oligomers (marked with a blue rectangle, for example) and larger protofibril forms (marked with red arrows) (Figure 3e; the high-resolution image is presented in Supplementary Figure S2). 

**Figure 4:**
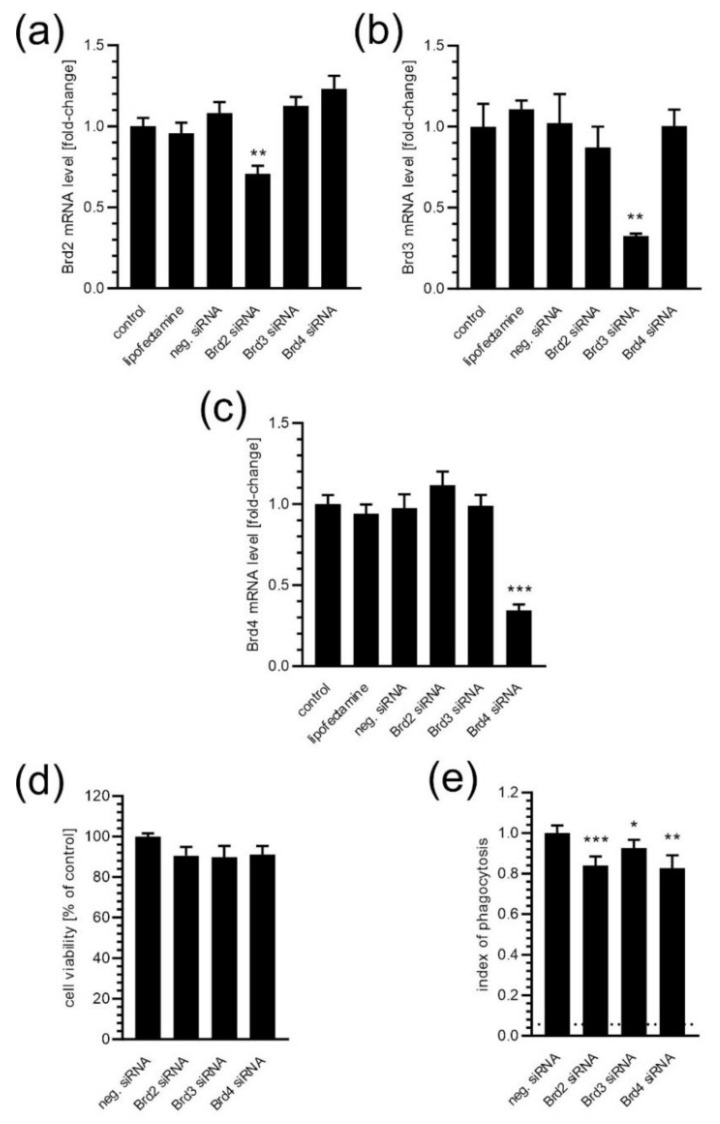
The effect of BET silencing on fluorescent microspheres’ (FMSs) phagocytosis by microglia. (**a**–**c**) mRNA levels for *Brd2*, *Brd3*, *Brd4* genes 24 h after gene silencing. (**d**) The effect of *Brd2*, *Brd3*, and *Brd4* gene silencing on viability BV2 cells (MTT assay). (**e**) The BV2 cells were subjected to gene silencing. After 24 h, FMS were added for 2 h. Cells were stained with blue nuclear dye and flow cytometric analysis was performed. The level of non-specific labelling is shown with a dotted line. Means ± SEM are presented. *n* = 8 (**a**–**c**), *n* = 4 (**d**), *n* = 12 (**e**); * *p* < 0.05, ** *p* < 0.01, *** *p* < 0.001, compared to the negative siRNA-treated group. Statistical analysis was performed by using a one-way ANOVA followed by the Bonferroni post hoc test.

To analyse which isoform of BET proteins is involved in controlling the phagocytic function of microglia, the gene silencing method was used. As demonstrated in Figure 4a–c, siRNA evoked an efficient and specific decrease in mRNA levels of all three brain-expressed isoforms of BET, *Brd2*, *Brd3*, and *Brd4*, and this siRNA treatment did not affect BV2 cells’ viability (Figure 4d). The silencing of each isoform evoked a moderate inhibitory effect on microglial phagocyting activity, with the prevalent effect observed for *Brd2* and *Brd4* (Figure 4e).

**Figure 5:**
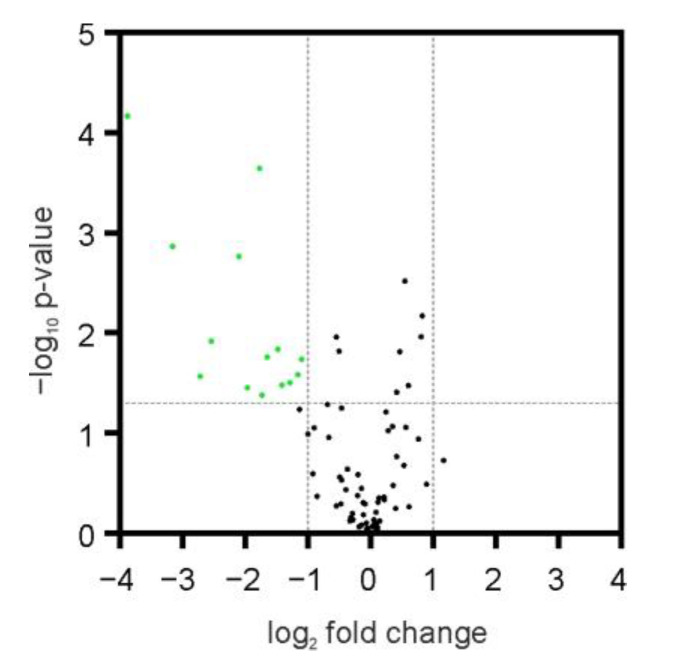
The effect of BET inhibitor JQ1 on the expression of phagocytosis-related genes in phagocyting microglia. BV2 cells were incubated in the absence or presence of 50 nM JQ1 for 24 h, and then FMS were added for 2 h and mRNA levels were determined using a gene expression array. The genes, the expression of which was significantly (FC > 2, *p* < 0.05) altered by JQ1, were marked on the volcano plot with green color. *n* = 4. Statistical analysis was performed by using the Student *t*-test.

Because the mechanism of BET inhibition-related changes in phagocytic activity of microglia is likely evoked by alterations in gene expression, we performed the real-time PCR screening experiment to identify the phagocytosis-associated genes that were affected by JQ1 treatment. We used a gene expression array that included a panel of 84 target and 12 control genes. Among those, the expression of 75 was detected in BV2 cells. For detailed information about this analysis, please see Supplementary Table S1. In this experiment, we pre-treated BV2 cells with 50 nM JQ1 for 24 h and subsequently induced phagocytosis by 2 h exposition to FMS, and observed that the mRNA level of 14 genes was significantly reduced (fold change (>2, *p* < 0.05) in phagocyting JQ1-pretreated BV2 cells (Figure 5). No gene expression was elevated by JQ1. The mRNA level of six genes (*Siglec1*, *Sirpb1a*, *Cd36*, *Clec7a*, *Itgam*, *Tlr3*) was found to be the most severely decreased; therefore, we have selected those for further detailed analysis.

**Figure 6:**
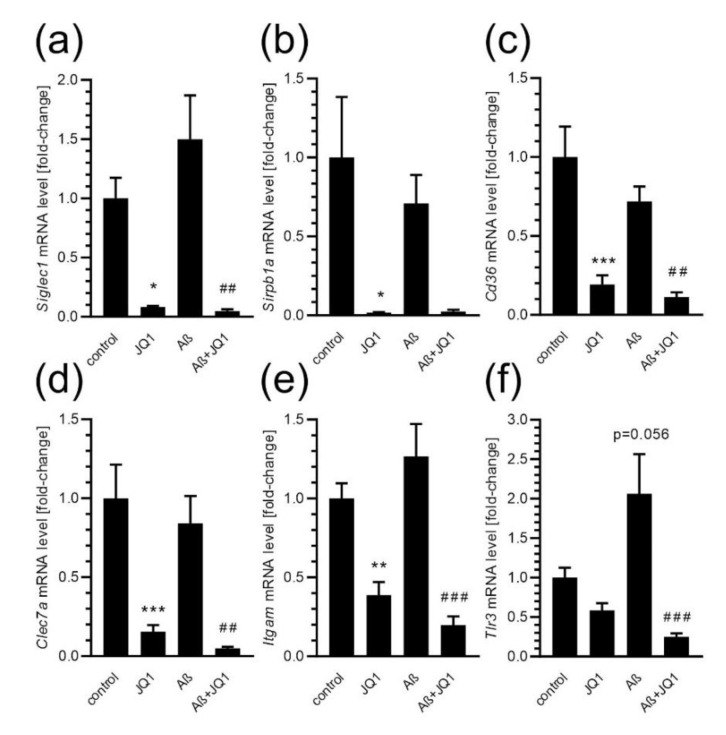
The effect of the BET inhibitor on microglial expression of phagocytosis-related genes. BV2 cells were incubated in the absence or presence of JQ1 for 24 h, and then Aβ was added for 2 h and mRNA levels for *Siglec1* (**a**), *Sirpb1a* (**b**), *Cd36* (**c**), *Clec7a* (**d**), *Itgam* (**e**), and *Tlr3* (**f**) were measured using the qPCR method. Means ± SEM are presented. *n* = 6–8; * *p* < 0.05, ** *p* < 0.01, *** *p* < 0.001, compared to control, ##, ### *p* < 0.01 and *p* < 0.001, compared to Aβ group. Statistical analysis was performed by using a one-way ANOVA followed by the Bonferroni post hoc test.

Subsequently, the impact of JQ1 on *Siglec1*, *Sirpb1a*, *Cd36*, *Clec7a*, *Itgam*, and *Tlr3* expression after Aβ treatment of BV2 cells was investigated. As shown in Figure 6, in our experimental conditions, Aβ did not significantly affect the mRNA level of investigated genes, although a weak upward trend in the *Tlr3* expression was observed. BET inhibition efficiently down-regulated the mRNA level of all tested genes in control conditions and/or after stimulation with Aβ.

**Figure 7:**
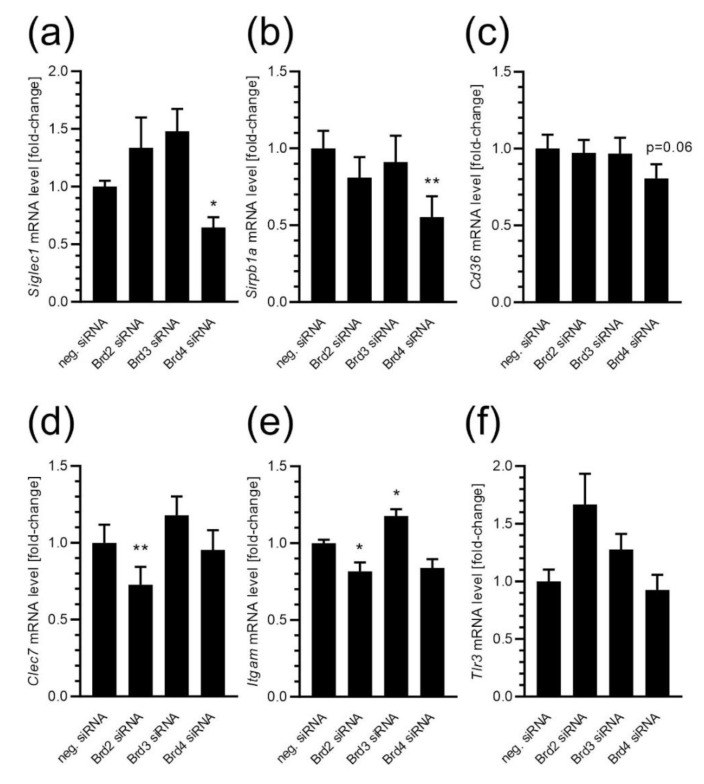
The effect of BET silencing on the microglial expression of phagocytosis-related genes in BV2 cells. The mRNA levels for *Siglec1* (**a**), *Sirpb1a* (**b**), *Cd36* (**c**), *Clec7a* (**d**), *Itgam* (**e**), and *Tlr3* (**f**) were determined 24 h after gene silencing by using the qPCR method. Means ± SEM are presented. *n* = 8; * *p* < 0.05, ** *p* < 0.01, compared to the negative siRNA-treated group. Statistical analysis was performed by using a one-way ANOVA followed by the Bonferroni post hoc test.

To reveal which BET isoform is involved in the observed phenomena, we performed silencing of particular isoforms and analysed the expression of selected phagocytosis-related genes. As shown in Figure 7, the silencing of *Brd2* decreased mRNA levels of *Clec7* and *Itgam*, and the silencing of *Brd4* evoked a decline in *Siglec1* and *Sirpb1a* expression. Remarkably, silencing of *Brd3* seemed to not affect the expression of selected genes, except for *Itgam*, which was significantly elevated upon *Brd3* down-regulation. 

Finally, we examined the effect of BET inhibitor JQ1 on mRNA levels of phagocytosis-related genes which were previously demonstrated to be involved in the pathomechanism of Alzheimer’s disease: *Abca7*, *Bin1*, *Cd2ap*, *Cd33*, *Clu*, *Cr1l* (the murine ortholog of human *CR1)*, *Picalm*, *Rab10*, *Rin3*, *Scara3*, *Trem2*, and *Zyx*. As shown in Table 1, 24 h of incubation in the presence of BET inhibitor JQ1 significantly decreased the expression of *Cd33*, *Trem2*, and Zyx. The levels of mRNA for *Abca7*, *Bin1*, *Cd2ap*, *Clu*, *Cr1l*, *Picalm*, *Rab10*, *Rin3*, and *Scara3* were not affected by JQ1.

## 3. Discussion

Microglial phagocytosis plays an important role in the development, health, and disease of the CNS. During development, microglia phagocytose excessive synapses, dendrites, axons, and neurons. To maintain a healthy brain, microglia phagocytose invading pathogens, dying or dead cells, cellular debris, or protein aggregates. Microglia may also contribute to neurodegeneration by excessive phagocytosis of live synapses or even stressed-but-viable neurons [2,7,8,26]. In our study, we showed for the first time that BET proteins control the phagocytic activity of microglia. We used immortalized murine microglial BV2 cell line, which is frequently used as a substitute for primary microglia due to similar antigen patterns, and phagocytic and cytotoxic activity [27,28]. However, this might state the major limitation of this study, since some differences between human and murine microglia, including changes in gene expression, were observed [29]. Transcriptomic analysis demonstrated that genes expressed by human and murine microglia were similar, but a limited overlap between humans and mice was found in microglial genes regulated during aging [30]. However, in our opinion, despite considerable differences between human and rodent physiology, molecular processes are similar, and using murine cell lines may provide a good introduction to the investigations on human-derived cells. To investigate the role of BET proteins in the processes of phagocytosis regulation, we used either gene silencing of specific isoforms or pharmacological inhibition. Although many proteins (over forty) contain a bromodomain in their structure, the proteins of the BET family (Brd2, Brd3, Brd4, and BrdT) have a unique domain architecture: they contain two conserved amino-terminal bromodomains that recognize acetylated lysine residues and a divergent carboxy-terminal recruitment domain. Because BrdT is not expressed in the brain, we focused our study on Brd2, Brd3, and Brd4. JQ1, a pan-inhibitor of the BET family, was used to inhibit all BET proteins [31]. JQ1 binds directly to the Kac (acetylated lysine) binding site in both bromodomains of BET proteins, but no significant binding was detected for bromodomains in proteins outside the BET family, suggesting that JQ1 is highly selective inhibitor of Brd2, Brd3, and Brd4 [31]. Therefore, to investigate the role of specific BET isoforms, we had to perform additional experiments using the gene silencing technique. Previous studies demonstrated the significant contribution of BET in regulating the expression of many genes related to immune system function; however, the role of those proteins in regulating phagocytosis has never been tested. In this study, we observed significant changes in the expression of phagocytosis-associated genes and a reduction in phagocytosis in BV2 cells after pharmacological or genetic inhibition of BETs. Our results showed that the pan-inhibitor of BET proteins, JQ1, at a low 50 nM concentration, which was demonstrated to be effective and non-toxic, significantly decreased phagocytosis in microglial cells. The effect of the BET inhibitor was comparable to the one observed for the inhibitor of phagocytosis, CCHD. Moreover, the application of the low concentration of the inhibitor enabled avoidance of its possible off-target action, which might appear at higher concentrations. 

The previous studies analysed other aspects of BET-controlled phagocytosis in dendritic cells. Riganti et al. demonstrated that the effect of inhibitors of BET proteins, JQ1 and OTX-015, may be dependent on phagocyted cell type [32]. Authors demonstrated that after prolonged (6 days) incubation in the presence of 250 nM JQ1, the malignant pleural mesothelioma (MPM) cells, but not normal non-transformed mesothelial cells (HMC) displayed elevated expression of the “eat-me” signals, calreticulin and ERp57. Consistently, JQ1 pre-treatment had no effect on dendritic cell-mediated phagocytosis of HMC, but increased phagocytosis of MPM cells [32]. Another study focusing on the effect of JQ1 on macrophage-mediated phagocytosis of melanoma cells demonstrated an attenuation of signal regulatory protein α (SIRP-α) expression and an increase in phagocytosis [33,34]. SIRP-α is a receptor that interacts with a transmembrane protein CD47 expressed in phagocytosed cells and is known as the “do not eat me” signal. Therefore, in the above studies, inhibition of BET could affect gene expression in either phagocytosing (dendritic cells, macrophages) or phagocytosed cells (MPM, HMC, melanoma), and thus might alter the interplay between them. In our experimental model, we studied microglial cells that phagocytose fluorescent microspheres or Aβ. Therefore, inhibition of BET proteins could only affect the cellular machinery in the phagocytosing cells. This could explain why the effect of JQ1 on the phagocytosis of cells and non-cellular material (in our study) is different. An additional difference between that and our study, which might impact the result of JQ1 action, is the concentration of JQ1: 1–2.5 µM versus 50 nM, respectively. Additionally, in the study of Benham and co-workers, the high 5 µM concentration of another BET inhibitor, I-BET, was demonstrated to significantly up-regulate the expression of genes involved in phagocytic processes, thus elevating phagocytic activity of murine bone marrow-derived macrophages [35]. In our study, we demonstrated that attenuation of BET proteins decreased the expression of many genes related to the phagocytic pathway in BV2 cells. Consistently, phagocytic activity reduced by JQ1 treatment was confirmed by using the gene silencing of particular *Brd* isoforms. 

The mechanism of BET protein activity is predominantly connected with the regulation of gene transcription; thus, the inhibition of those proteins is likely associated with changes in the expression of genes related to phagocytic processes. Our array analysis of mRNA levels of 84 phagocytosis-related genes identified 14 JQ1-sensitive genes that were significantly down-regulated. However, we assumed the changes in the gene expression that were higher than two-fold to be significant. We also observed that the mRNA level of some genes was significantly (*p* < 0.05) up- or down-regulated, but those changes were less than two-fold; thus, they might play a minor role in JQ1-induced phagocytosis attenuation. Interestingly, we have observed that various BET isoforms regulated the expression of phagocytosis-related genes in a different manner: while the Brd2 and Brd4 seemed to be involved in the stimulation of the above-mentioned genes, the Brd3 rather inhibited their expression. This is in agreement with the previous studies, suggesting overlapping but distinct roles for individual BET proteins in metabolic regulation [36,37]. Because of the diverse specificity of JQ1 against various BET isoforms [38,39,40,41], it can be expected that different JQ1 concentrations may change the expression pattern of various phagocytosis-related genes in a different way. It was previously demonstrated that various BET isoforms function jointly, but may also play separate roles in gene transcription. For example, in Th17 cells, over 90% of Brd4-associated genes were also connected with Brd2, but over 70% of Brd2 target genes did not overlap with target genes of Brd4 [24]. It is also possible that some effects of BET inhibitors could be evoked by BET’s function that is not directly attributed to their role as acetylation code readers. Interestingly, BET family proteins have been identified as atypical kinases, which might affect gene transcription also by alternative mechanisms [42]. For example, Brd4 may affect gene transcription by phosphorylation of transcription machinery [43]. Brd4 possesses intrinsic kinase activity and may directly phosphorylate RNA polymerase II, TATA-box binding protein associated factor 7 (TAF7), and positive transcription elongation factor b/cyclin-dependent kinase 9 (PTEFb/CDK9) [44,45]. Moreover, Brd4 may also phosphorylate transcription factor MYC (master regulator of cell cycle entry and proliferative metabolism) at Thr58, leading to its ubiquitination and degradation [46].

The role of microglia in AD pathology is complex and not fully understood. The early studies stimulated the conception of the “autotoxic loop” [47]. According to this theory, activation of microglia by primary disease-associated factors leads to the release of several neurotoxic cytokines and other compounds which accelerate neurodegeneration and evoke the release and accumulation of cellular debris that, in consequence, may reinforce microglial activation. This concept of the detrimental role of “pro-inflammatory” activation of microglia was supported by epidemiologic studies which demonstrated that long-lasting use of non-steroid anti-inflammatory drugs reduces the risk of developing AD [48,49]. However, several studies which used genetic manipulation to modulate inflammatory pathways in animal models of AD demonstrated high variability in the results, indicating the complex role of microglia and neuroinflammation in the pathomechanism of AD [50]. The most important role of microglia in AD seems to be related to its beneficial phagocytic activity towards Aβ and to the release of detrimental pro-inflammatory cytokines; therefore, promoting microglial phagocytosis together with mitigating excessive inflammatory response was proposed to be a promising therapeutic strategy [51]. 

Recent studies highlighted the possibility that microglia may contribute to the spreading of Aβ pathology in AD. For example, it was demonstrated that microglia might directly contribute to amyloid plaque formation. Microglial depletion, which was evoked in 5×FAD mice by a selective brain-penetrant CSF1R inhibitor (PLX5622), prevented amyloid plaque formation in the brain parenchyma [52]. It was also recently suggested that microglia build plaques rather than remove them [53]. Authors demonstrated in a transgenic model of amyloidosis, mice APP/PS1, that impaired microglial phagocytosis results in the development of fewer dense-core plaques. The intriguing hypothesis of confinement mechanism was proposed: microglia might limit the dissemination of toxic Aβ oligomers in the brain by taking them up, compacting them in the acidic environment of the lysosomes, and finally releasing that less toxic material, and therefore contributing to building dense-core plaques. 

Recent genome-wide association studies (GWAS) confirmed the previous concept that microglial phagocytosis may play a significant role in the pathomechanism of AD. These GWAS studies demonstrated that polymorphism of several phagocytosis-related genes, for example, *TREM2*, *PICALM*, *BIN1*, *CD2AP*, and *ABCA7*, increases the risk of developing AD [54,55,56]. Our analysis of established genetic risk factors of AD and other phagocytosis-related genes involved in the pathomechanism of AD demonstrated that among tested genes, JQ1 significantly reduced the expression of *Cd33*, *Trem2*, and *Zyx*. CD33, a member of the SIGLEC (sialic acid-binding immunoglobulin-type lectins) family, is a phagocytic receptor that was implicated in the pathomechanism of AD as a modifier of Aβ pathology. The increased CD33 (mRNA and protein) levels in the AD brains were observed, and CD33 promoted Aβ deposition and plaque formation in vivo [57]. In circulating monocytes of patients bearing the rs3865444(C) risk allele, alternative splicing of exon 2 leads to higher cell surface expression of CD33 [58]. This, in turn, increased microglial activation, enhanced accumulation of Aβ, and evoked deterioration of cognitive functions [59]. The presence of the minor rs3865444(T) protective allele was associated with reduced CD33 protein levels and also with reduced levels of insoluble Aβ in the AD brain [57]. Additionally, the inactivation of CD33 in transgenic mice *APP_Swe_/PS1_ΔE9_* reduced brain levels of insoluble Aβ42 as well as mitigated Aβ plaque pathology [57]. The mechanism of CD33-related microglial Aβ uptake was dependent on interaction with sialic acid [57]. Collectively, the data suggest that CD33, as a regulator of microglial clearance of Aβ, may be a relevant target for the treatment and prevention of AD. Our data thus indicate that BET protein modulation might be an indirect way by which the activity of CD33 might be down-regulated, thus slowing down the AD progression. Moreover, polymorphism of *CD33* was suggested to impact the pathomechanism of multiple sclerosis [60] and Parkinson’s disease [61].

ZYX (zyxin; previously EPHA1) is a LIM domain adaptor protein that is involved in many cellular processes, including signal transduction and modulating gene expression, but also interaction of the cell with the extracellular matrix, and organisation and function of the cytoskeleton, including endocytosis [62]. It was suggested that zyxin is associated with actin filaments and participates in actin remodelling and active engulfment [63,64]. Moreover, zyxin has the pathological relevance in carcinogenesis as a regulator of the homeodomain-interacting protein kinase 2 (HIPK2)-p53 signalling axis in response to DNA damage [65,66,67]. In AD, the expression of zyxin is reduced, and the role of zyxin is also not limited to regulating endocytosis [68]. In vitro studies in neuronal SH-SY5Y cells demonstrated that zyxin was attenuated by intracellular Aβ peptides, leading in consequence to the deregulation of the HIPK2-p53 pathway [69]. Moreover, zyxin was proposed to be a suitable neuron-derived exosomal protein marker in serum, whose expression drops before a clinical diagnosis of AD [68]. Considering that zyxin is down-regulated in AD, attenuation of *Zyx* expression by inhibiting BET might not have beneficial effects, but this question requires further research.

TREM2 (triggering receptor expressed on myeloid cells 2), a member of the immunoglobulin superfamily, is a pattern recognition receptor highly expressed in microglia. TREM2 recognizes a wide array of ligands, including bacterial compounds (LPS), DNA, phospholipids, glycolipids, lipoproteins, apolipoproteins, and also Aβ. In the brain, TREM2 is selectively expressed in microglia, and its stimulation activates transcriptional changes, phagocytosis, chemotaxis, and other processes related to the activation of the innate immune response [70]. TREM2 plays an important role in the pathomechanism of AD; some loss-of-function mutations of the *TREM2* gene showed a high association with AD prevalence. For example, TREM2 gene variant rs75932628(T) increased by two to three times the risk for AD in European and North American populations [71]. In the brains of AD patients, TREM2 interacts with components of senile plaques, including Aβ, lipo-, and apolipoproteins [70]; and the elevation of soluble TREM2 in cerebrospinal fluid (CSF) negatively correlated with plaque growth, cortical shrinkage, and cognitive decline [72]. Experimental TREM2 deletion gives conflicting results, showing a decrease or increase in amyloid pathology, depending on the model employed, age, and brain region [73]. It seems that the outcome of TREM2 function may be protective in the early stages of AD and detrimental in the late phase of the disease [63,73]. Activation of TREM2 signalling in transgenic AD mice models using humanized monoclonal IgG1 agonistic antibody AL002 normalized behavior [74], and this antibody successfully underwent phase I clinical trials (NCT03635047) and is currently in phase II (NCT04592874), which involves participants with early AD. Considering that the role of TREM2 changes during the development of AD, being positive in the early stages and negative in the late stages, down-regulation of *Trem2* expression by the inhibition of BET family proteins might have a beneficial effect only in the late stage of AD. However, confirmation of this phenomenon requires further research.

In summation, our study demonstrated that BET proteins play an important role in controlling microglial function and BET’s inhibition may significantly affect microglial phagocytosis, and thus may potentially open up new avenues for developing novel therapeutic strategies. This is especially interesting since some BET inhibitors are now undergoing clinical trials as therapeutics for several disorders, including cancer. Therefore, understanding the effect of BET inhibitors on microglial phagocytosis should be critically important for ongoing and future therapeutic strategies for AD.

## 4. Materials and Methods

### 4.1. Chemicals

Reagents for reverse transcription (High-Capacity cDNA Reverse Transcription Kit with RNase Inhibitor) and quantitative PCR (Taqman Assays, TaqMan OpenArray Mouse Phagocytosis Panel, and TaqMan Fast Advanced Master Mix), red fluorescent microspheres (FMS), 2.0 μm, Hoechst 33342, CellMask Orange Actin Tracking Stain, CellMask Green Actin Tracking Stain, RPMI were obtained from Thermo Fisher Scientific, Inc. (Waltham, MA, USA). Opti-MEM was from Gibco, negative siRNA control was from Ambion, Lipofectamine was from Thermo Fisher Scientific, Inc., Aβ_1–42_ and Aβ_1–42_ HiLyte 488 were from AnaSpec, Inc. (Fremont, CA, USA). JQ1, GSK12101517, IBET-762, OTX-015, PFI-1, Cytochalasin D were obtained from Sigma-Aldrich (St. Louis, MO, USA). Heat-inactivated foetal bovine serum (FBS), Accutase solution, penicillin, streptomycin, L-glutamine, 3-(4,5-dimethyl-2-tiazolilo)-2,5-diphenyl-2H-tetrazolium bromide (MTT), propidium iodide, TRI-reagent, DNase I, dithiothreitol (DTT), anhydrous dimethyl sulfoxide (DMSO), lipopolysaccharide from *Escherichia coli* O55:B5 (toxicity 3,000,000 U/mg), and all other reagents were obtained from Sigma-Aldrich (St. Louis, MO, USA). 

### 4.2. Cell Culture 

Murine microglial BV2 cells were purchased from Elabscience Biotechnology Inc. (Houston, TX, USA) [75]. The cells were cultured in RPMI supplemented with 10% FBS, 2 mM L-glutamine, 50 units/mL penicillin, and 50 μg/mL streptomycin in 5% CO_2_ atmosphere at 37 °C. The passages below 20 were used for experiments. The cells were regularly tested to exclude mycoplasma contamination.

JQ1, other BET inhibitors, and Cytochalasin D (CCHD) were dissolved in DMSO at 10 mM stock solution and then were diluted with a culture medium and added to cells at a specified concentration. The final DMSO concentration was 0.1%. Lipopolysaccharide (LPS) was dissolved in saline. In all experiments, the respective vehicle was added to corresponding groups accordingly.

### 4.3. Silencing of Bet Proteins Expression

Gene silencing protocol was adopted from our previous study [76]. Shortly, BV2 cells were seeded on a six-well plate at a density of 3.8 × 10^4^/cm^2^ in 2.5 mL of RPMI medium supplemented with 10% FBS and 2 mM L-glutamine. Immediately, the 10 min preincubated mixture of 500 µL Opti-MEM with 2.5 µL lipofectamine and 20 µL siRNA for Brd2, Brd3, Brd4, or the negative control was added dropwise. Then, the cells were cultured in standard conditions for 24 h.

### 4.4. Determination of Cell Survival (MTT Reduction Assay) and Cytotoxicity (PI Uptake Assay)

Cellular viability was evaluated by the reduction of 3-(4,5-dimethyl-2-tiazolilo)-2,5-diphenyl-2H-tetrazolium bromide (MTT) to formazan. After treatment with investigated compounds, MTT (0.25 mg/mL) was added and cells were incubated at 37 °C for the next 2 h. The medium was removed, the cells dissolved in DMSO, and the absorbance of formazan was measured at 595 nm using a Multiskan GO Microplate Spectrophotometer (Thermo Fisher Scientific, Inc.).

Cytotoxicity was evaluated by using propidium iodide (PI) staining. After treatment with investigated compounds, PI (8 µM) was added to a culture medium, and cells were incubated in the dark at room temperature for 20 min. Then, cells were collected and subjected to flow-cytometric analysis on a FACS Canto II cytometer (BD Biosciences, San Jose, CA, USA). 

### 4.5. Determination of Microglial Proliferation

For analysis of cell proliferation, equal numbers of cells were seeded on a 6-well plate. After 24 h, 50 nM JQ1 or a respective vehicle were added and incubation was continued for the next 24 h. Then, cells were detached and counted by using Cell Counter (Bio-Rad, Hercules, CA, USA).

### 4.6. Analysis of Microglial Migration (Scratch Assay)

Microglial migration was analysed with the scratch assay [77]. Murine microglial BV2 cells were seeded at a density of 3.8 × 10^4^/cm^2^. After 24 h, a single layer of cells was scraped in a straight line to create a “scratch” with the tip of the pipette. The floating cells were removed by washing twice with warm PBS, and then RPMI, with reduced FBS concentration (2%), to attenuate proliferation, was added and cells were incubated in standard conditions in the presence of 50 nM JQ1 for 24 h. Then, pictures were taken under the microscope and cells in the “scratch area” were counted by a blinded operator using ImageJ software (ver. 1.52p; National Institutes of Health, Bethesda, MD, USA; https://imagej.nih.gov/ij/) [78]. 

### 4.7. Determination of Phagocytic Activity of Microglia (Fluorescent Microspheres Assay)

Microglial phagocytosis was analysed by flow cytometry and confocal microscopy [25,79,80]. For flow cytometry, BV2 cells were seeded to a 12-well plate at a density of 3.2 × 10^4^ cells/cm^2^. After 24 h, cells were incubated in the presence of a tested compound in standard conditions, and then FMS (0.5 × 10^6^/mL) were added and incubation was continued for the next 2 h. Then, cells were washed three times with PBS to remove the non-phagocyted FMS and incubated in Accutase solution supplemented with and Hoechst 33342 (2 µg/mL) for 15 min in the dark at 37 °C. Cells were collected, and the presence of FMS in living microglial cells was detected by flow cytometry using BD FACS Canto II and DIVA 6 software (BD Biosciences, San Jose, CA, USA). The Hoechst-positive BV2 cell population was gated and the number of FMS-labelled cells was measured. Cytochalasin D (CCHD; 2 µM) was used as an inhibitor of phagocytosis. To evaluate the level of non-specific binding of FMS to cells, the incubation was performed at 4 °C. The index of phagocytosis was calculated as follows: index = % of FMS-labelled cells in analysed group/% of FMS-labelled cells in the control.

For microscopic confocal analysis, BV2 cells were seeded on Cellview Cell Culture Slides (Greiner) in a medium with reduced FBS concentration (2%) to attenuate proliferation. After 24 h, 50 nM JQ1 was added for the next 24 h. Then, Hoechst 33342 (2 µg/mL) and Cell Mask Green Actin Tracker Stain (×1) were added, and cells were incubated in standard conditions. After 30 min, the fluorescent microspheres were added and incubation was continued for 2 h. Then, cells were analysed under a confocal microscope.

### 4.8. Analysis of the Aβ Uptake by Microglial Cells Using Fluorescent Aβ HiLyte488

Aβ uptake was analysed by flow cytometry and confocal microscopy, as described previously [80]. Directly before analysis, HFIP-pretreated Aβ_1–42_ HiLyte 488 was dissolved (5 mM) in anhydrous DMSO and further diluted in a cell culture medium to 100 µM concentration. After 30 s vortexing, Aβ preparations were directly used for cell treatment.

For flow cytometry, BV2 cells were seeded to a 12-well plate at a density of 3.2 × 10^4^ cells/cm^2^ for 24 h. Then, incubation was continued in the presence of JQ1 (50 nM) for 24 h or with Cytochalasin D (CCHD; 2 µM) for 30 min. Aβ HiLyte488 (1 µM) was added for the next 2 h. Then, Hoechst 33342 (2 µg/mL) and Cell Mask Orange Actin Tracker Stain (×1) were added, and cells were incubated in standard conditions for 30 min. The presence of Aβ in living microglial cells was detected by flow cytometry using BD FACS Canto II and DIVA 6 software (BD Biosciences). The Hoechst-positive BV2 cell population was gated and the mean fluorescence intensity in cells within the gate was measured. To determine the level of non-specific binding of Aβ HiLyte488 with the cell surface, the additional group was incubated at 4 °C. 

For microscopic confocal analysis, BV2 cells were seeded on Cellview Cell Culture Slides (Greiner) in a medium with reduced FBS concentration (2%) to attenuate proliferation. After 24 h, 50 nM JQ1 was added for the next 24 h. Then, Hoechst 33342 (2 µg/mL) and Cell Mask Orange Actin Tracker Stain (×1) were added, and cells were incubated in standard conditions. After 30 min, Aβ_1–42_ HiLyte 488 (1 µM) was added and incubation was continued for 2 h. Then, cells were analysed under the confocal microscope.

### 4.9. Analysis of Gene Expression

RNA was isolated by using TRI reagent (Sigma-Aldrich) according to the manufacturer’s protocol. The concentration and quality of RNA were measured using a Nanodrop spectrophotometer (Thermo Fisher Scientific, Inc.). Digestion of potential DNA contamination was performed by using DNase I, according to the manufacturer’s protocol (Sigma-Aldrich). Reverse transcription was performed using a High-Capacity cDNA Reverse Transcription Kit with RNase Inhibitor according to the manufacturer’s protocol (Thermo Fisher Scientific, Inc.). 

#### 4.9.1. Gene Expression Array Plates

The level of mRNA for phagocytosis-related genes was analysed using TaqMan OpenArray Mouse Phagocytosis Panel (Catalog number: 4471126) according to the manufacturer’s protocol (Thermo Fisher Scientific, Inc.). An amount of 40 ng of cDNA was used per each well. Data were analysed and calculated with Expression Software version 1.3 (Thermo Fisher Scientific, Inc.) using global normalization and the relative levels of mRNA were calculated using the ΔΔCt method.

#### 4.9.2. TaqMan Assays

The level of mRNA for selected genes was analysed using TaqMan Gene Expression Assays (Thermo Fisher Scientific, Inc.): *Actb* (Mm04394036_g1), *Gusb* (Mm01197698_m1), *Hprt* (Mm00446968_m1), *Il1b* (Mm00446190_m1), *Abca7* (Mm00497010_m1), *Apoe* (Mm01307193_g1), *Bin1* (Mm00437457_m1), *Cd2ap* (Mm00815310_s1), *Cd33* (Mm00491152_m1), *Cd36* (Mm01135198_m1), *Clec7a* (Mm01183349_m1), *Clu* (Mm01197002_m1), *Cr1* (Mm00785297_s1), *Itgam* (Mm00434455_m1), *Picalm* (Mm00525455_m1), *Rab10* (Mm00489481_m1), *Rin3* (Mn00617220_m1), *Scara3* (Mm00553769_m1), *Siglec1* (Mm00488332_m1), *Sirpb1* (Mm02525668_u1), *Tlr3* (Mm01207404_m1), *Trem2* (Mm04209424_g1), *Zyx* (Mm00496120_m1).

Quantitative PCR was performed on an Applied Biosystems 7500 Real-Time PCR System using TaqMan Fast Advanced Master Mix according to the manufacturer’s instructions. The relative levels of mRNA were calculated using the ΔΔCt method with *Gusb*, as a reference gene. To increase the validity and reproducibility of qPCR analysis in experiments with JQ1, the ΔΔCt calculation was extended by replacing the Ct of a single reference gene with an averaged Ct-value from three reference genes (*Actb*, *Gusb*, *Hprt*) [81].

### 4.10. Atomic Force Microscopy

AFM analysis was performed as described previously [82]. Shortly, amyloid samples were prepared by applying a drop of 10 μL medium on freshly cleaved mica, V1 grade (NanoAndMore GmbH, Germany). After incubation for 10 min, the sample was rinsed with deionised water (Merck Millipore Inc., Burlington, MA, USA) and dried under a gentle stream of argon. A Multimode 8 Nanoscope atomic force microscope (AFM, Bruker, Billerica, MA, USA) was used to image the surfaces of the mica substrate and the deposited amyloid structures. 

### 4.11. Statistical Analysis

The results were expressed as mean values ± SEM. The statistical analysis of the data was performed by using GraphPad Prism version 8.3.0 (GraphPad Software, San Diego, CA, USA). The data distribution was analysed by using a normality test. The outliers were detected with the ROUT method. Data, depending on experimental design, were analysed using a Student *t*-test or a one-way analysis of variance (ANOVA) with Bonferroni post hoc test with correction for multiple comparisons; *p* values < 0.05 were considered significant. The *n* number refers to independent experiments.

## Figures and Tables

**Table 1:** The effect of the BET inhibitor on microglial expression of phagocytosis-related AD-involved genes.

	mRNA Level [Fold-Change]		
Gene	Control	JQ1	
*Abca7*	1.00 ± 0.09	1.13 ± 0.08	
*Apoe*	1.00 ± 0.30	1.38 ± 0.33	
*Bin1*	1.00 ± 0.11	1.08 ± 0.10	
*Cd2ap*	1.00 ± 0.08	1.24 ± 0.11	
*Cd33*	1.00 ± 0.17	0.17 ± 0.04	***
*Clu*	1.00 ± 0.10	1.23 ± 0.14	
*Cr1l*	1.00 ± 0.11	1.04 ± 0.11	
*Picalm*	1.00 ± 0.05	1.01 ± 0.02	
*Rab10*	1.00 ± 0.06	1.23 ± 0.09	
*Rin3*	1.00 ± 0.09	1.27 ± 0.13	
*Scara3*	1.00 ± 0.11	0.87 ± 0.11	
*Trem2*	1.00 ± 0.11	0.45 ± 0.06	***
*Zyx*	1.00 ± 0.11	0.61 ± 0.05	**

BV2 cells were incubated in the absence or presence of JQ1 for 24 h. Then, mRNA levels were measured using the qPCR method. Means ± SEM are presented. *n* = 6–8; ** *p* < 0.01, *** *p* < 0.001, compared to the control. Statistical analysis was performed by using the Student *t*-test.

## Data Availability

The data presented in this study are available on request from the corresponding author.

## Supplementary Materials

The supplementary materials are available online at https://www.mdpi.com/article/10.3390/ijms24010013/s1.
