# Supplementary material for: The Role of Bromodomain and Extraterminal (BET) Proteins in Controlling the Phagocytic Activity of Microglia In Vitro: Relevance to Alzheimer’s Disease
Source: Int J Mol Sci. 2022 Dec 20;24(1):13. doi: 10.3390/ijms24010013 (PMC9820364; doi:10.3390/ijms24010013)
Supplement: Supplementary file 1 [file ijms-24-00013-s001.zip › ijms-2057948-supplementary.pdf]

Supplementary materials

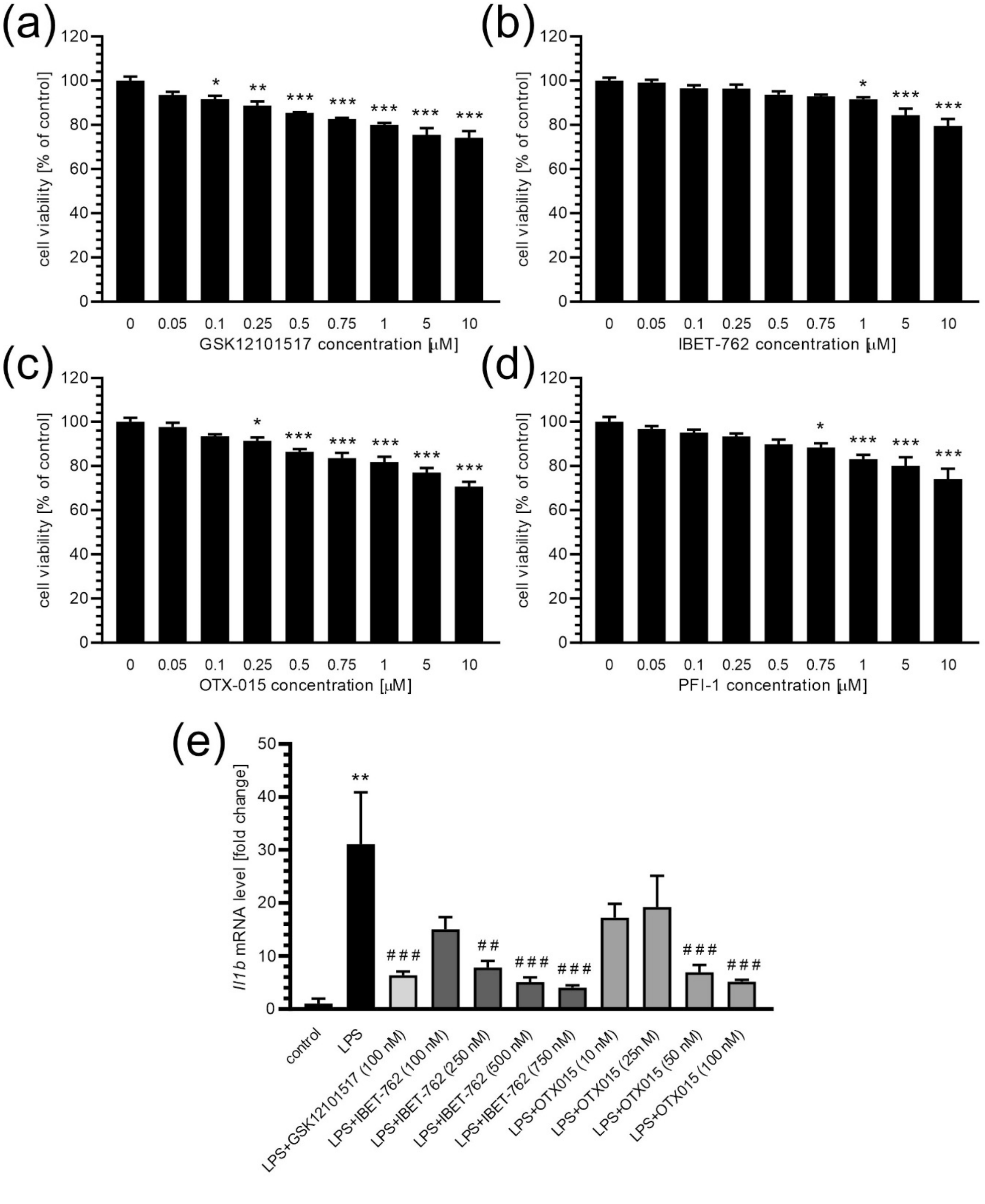

Supplementary Figure S1. The effect of BET inhibitors on microglial BV2 cells. (**a**–**d**) BV2 cells were incubated for 24 h in the presence of tested compounds, then MTT assay was performed to analyse cell viability (n=4). (**e**) BV2 cells were incubated for 2 h in the presence of LPS (100 ng/ml) and tested compound, then mRNA level for *Il1b* gene was determined by using qPCR (n=3-4). Means ± SEM were presented. *,**,*** $p<0.05$, 0.01, and 0.001, respectively, compared to the control. ##, ### $p<0.01$, and $p<0.001$, respectively, compared to LPS-treated group. Statistical analysis was performed by using a one-way ANOVA followed by the Bonferroni post-hoc test.

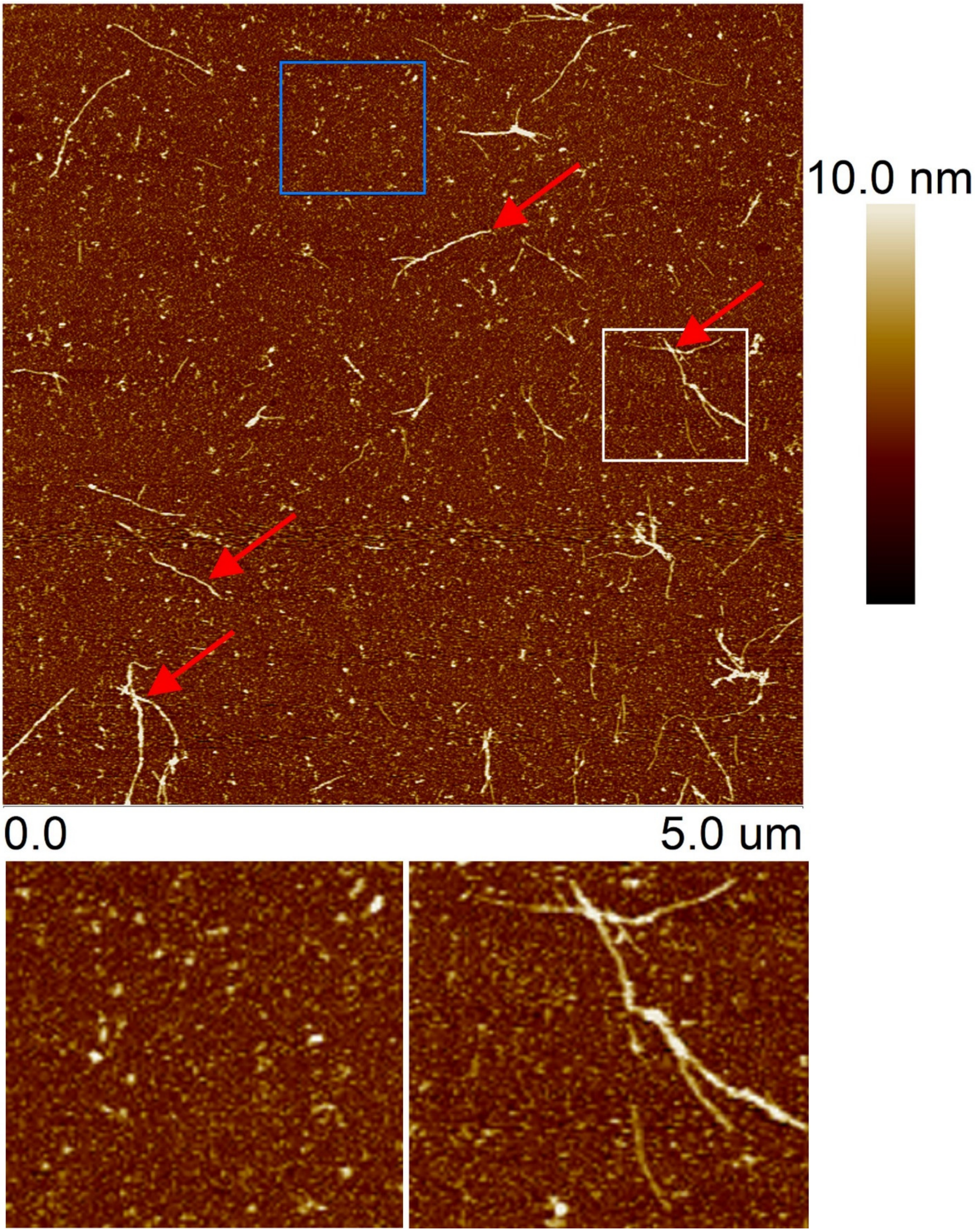

Supplementary Figure S2. High resolution AFM picture of Aβ after 2 h incubation at 37 °C. On the upper panel example oligomers were marked with a blue rectangle, red arrows indicate representative protofibrils. Lower panel shows marked areas in higher magnification.

Supplementary Table S1. The results of gene expression array analysis.

TaqMan Array Mouse Phagocytosis 96-well plate, fast, RPU62UA

Normalization method   Global Normalization

Fold Change Boundary 2.0

P-Value Boundary   0.05

Statistical Test   T-test

N=4

*   control

	Assay ID	Gene Symbol	Gene Name	Fold Change	P-Value	Result
*	Hs99999901_s1	18s rRNA	eukaryotic 18S rRNA	1.321	0.56012934	Insignificant
*	Mm00607939_s1	Actb	actin, beta	0.995	0.9702059	Insignificant
	Mm00456425_m1	Adipoq	adiponectin, C1Q and collagen domain containing	1.861	0.31970453	Insignificant
	Mm01134790_g1	Ager	advanced glycosylation end product-specific receptor	1.084	0.8682234	Insignificant
	Mm00440225_m1	Anxa1	annexin A1	0.411	0.031275354	Down-regulated
	Mm00437221_m1	Axl	AXL receptor tyrosine kinase	0.465	-	N.A.
*	Mm00437762_m1	B2m	beta-2 microglobulin	0.728	0.29210293	Insignificant
	Mm00437838_m1	C3	complement component 3	1.533	0.5392111	Insignificant
	Mm00482936_m1	Calr	calreticulin	0.960	0.9062711	Insignificant
	Mm00438094_g1	Cd14	CD14 antigen	0.301	0.041669343	Down-regulated
	Mm01135198_m1	Cd36	CD36 antigen	0.152	0.027002657	Down-regulated
	Mm01277163_m1	Cd44	CD44 antigen	0.501	0.10117423	Insignificant
	Mm00495011_m1	Cd47	CD47 antigen (Rh-related antigen, integrin-associated signal transducer)	0.975	0.886271	Insignificant
	Mm01257219_m1	Ceacam3	carcinoembryonic antigen-related cell adhesion molecule 3	-	-	N.A.
	Mm01183349_m1	Clec7a	C-type lectin domain family 7, member a	0.172	0.012091957	Down-regulated
	Mm01179940_mH	Clic4	chloride intracellular channel 4 (mitochondrial)	0.823	0.6299917	Insignificant
	Mm01169510_m1	Cnn2	calponin 2	-	-	N.A.
	Mm01236242_m1	Colec12	collectin sub-family member 12	0.827	0.72318345	Insignificant
	Mm00467065_m1	Crk	v-crk sarcoma virus CT10 oncogene homolog (avian)	1.071	0.8084952	Insignificant
	Mm00432680_g1	Crp	C-reactive protein, pentraxin-related	-	-	N.A.
	Mm00432686_m1	Csf1	colony stimulating factor 1 (macrophage)	0.537	0.08823264	Insignificant
	Mm01290062_m1	Csf2	colony stimulating factor 2 (granulocyte-macrophage)	-	-	N.A.
	Mm00432751_m1	Csk	c-src tyrosine kinase	1.042	0.7218633	Insignificant

	Mm00512037_m1	Cyp2s1	cytochrome P450, family 2, subfamily s, polypeptide 1	0.039	-	N.A.
	Mm01269874_m1	Dock1	dedicator of cytokinesis 1	0.985	0.9177907	Insignificant
	Mm00473720_m1	Dock2	dedicator of cyto-kinesis 2	1.021	0.8729215	Insignificant
	Mm00519109_m1	Elmo1	engulfment and cell motility 1	0.630	0.109474644	Insignificant
	Mm01204974_m1	Fas	Fas cell surface death receptor	0.886	0.85421735	Insignificant
	Mm02343757_m1	Fcer1g	Fc receptor, IgE, high affinity I, gamma polypeptide	0.777	0.22789556	Insignificant
	Mm00438874_m1	Fcgr1	Fc receptor, IgG, high affinity I	0.257	0.034961548	Down-regulated
	Mm00438875_m1	Fcgr2b	Fc receptor, IgG, low affinity IIb	0.376	0.03289869	Down-regulated
	Mm00438882_m1	Fcgr3	Fc receptor, IgG, low affinity III	0.457	0.05749123	Insignificant
	Mm00433373_m1	Fyn	FYN proto-oncogene, Src family tyrosine kinase	0.708	0.0152459815	Flat
*	Mm99999915_g1	Gapdh	glyceraldehyde-3-phosphate dehydrogenase	1.384	0.015334317	Flat
	Mm00518428_m1	Gulp1	GULP, engulfment adaptor PTB domain containing 1	-	-	N.A.
*	Mm00446953_m1	Gusb	glucuronidase, beta	1.042	0.8080083	Insignificant
*	Mm00660262_g1	Hmbs	hydroxymethylbilane synthase	2.248	0.1852197	Insignificant
*	Mm00446968_m1	Hprt	hypoxanthine guanine phosphoribosyl transferase	1.528	0.033382244	Flat
	Mm01168134_m1	Ifng	interferon gamma	-	-	N.A.
	Mm00516117_m1	Il1rl1	interleukin 1 receptor-like 1	0.722	0.5056077	Insignificant
*	Mm01255158_m1	Ipo8	importin 8	1.020	0.84822565	Insignificant
	Mm01182566_m1	Iqsec1	IQ motif and Sec7 domain 1	0.712	0.27312392	Insignificant
	Mm00434455_m1	Itgam	integrin alpha M	0.234	0.0017175004	Down-regulated
	Mm00434486_m1	Itgav	integrin alpha V	1.161	0.43396673	Insignificant
	Mm00434513_m1	Itgb2	integrin beta 2	0.926	0.6455412	Insignificant
	Mm01217488_m1	Lyn	Yamaguchi sarcoma viral (v-yes-1) oncogene homolog	1.191	0.060764432	Insignificant
	Mm01301009_m1	Mapk14	mitogen-activated protein kinase 14	1.486	0.087163374	Insignificant
	Mm00440265_m1	Marco	macrophage receptor with collagenous structure	0.320	0.017310344	Down-regulated
	Mm00487623_m1	Mbl2	mannose-binding lectin (protein C) 2	-	-	N.A.
	Mm00460328_m1	Mcoln3	mucolipin 3	0.685	0.528743	Insignificant
	Mm00434920_m1	Mertk	c-mer proto-oncogene tyrosine kinase	1.752	0.010825158	Flat
	Mm00500549_m1	Mfge8	milk fat globule-EGF factor 8 protein	0.868	0.4174912	Insignificant
	Mm03938638_s1	Mif	macrophage migration inhibitory factor	0.530	0.25248522	Insignificant
	Mm00447889_m1	Msn	moesin	0.871	0.2576329	Insignificant
	Mm00440338_m1	Myd88	myeloid differentiation primary response gene 88	1.108	0.7475546	Insignificant
	Mm00805062_m1	Nod1	nucleotide-binding oligomerization domain containing 1	0.467	0.018206742	Down-regulated
	Mm01242584_m1	Pecam1	platelet/endothelial cell adhesion molecule 1	0.906	0.82243997	Insignificant
*	Mm00435617_m1	Pgk1	phosphoglycerate kinase 1	1.025	0.8835571	Insignificant

	Mm00659576_m1	Pik3cb	phosphatidylinositol 3-kinase, catalytic, beta polypeptide	0.685	0.010959459	Flat
	Mm00476829_m1	Pip5k1a	phosphatidylinositol-4-phosphate 5-kinase, type 1 alpha	0.967	0.8899483	Insignificant
	Mm00447040_m1	Pla2g4a	phospholipase A2, group IVA (cytosolic, calcium-dependent)	0.730	0.056154333	Insignificant
	Mm00448162_m1	Pla2g5	phospholipase A2, group V	0.805	0.69393283	Insignificant
	Mm01289339_m1	Pld1	phospholipase D1	0.360	0.014450495	Down-regulated
	Mm00447066_m1	Pld2	phospholipase D2	0.958	0.78521127	Insignificant
	Mm00440894_m1	Prkce	protein kinase C, epsilon	1.090	0.48608992	Insignificant
	Mm01343426_m1	Pros1	protein S (alpha)	0.801	0.74985003	Insignificant
	Mm00477208_m1	Pten	phosphatase and tensin homolog	1.450	0.20915312	Insignificant
	Mm00727887_s1	Rab5a	RAB5A, member RAS oncogene family	0.924	0.49013537	Insignificant
	Mm01183732_g1	Rab7	RAB7, member RAS oncogene family	0.904	0.35396162	Insignificant
	Mm01201653_mH	Rac1	RAS-related C3 botulinum substrate 1	1.280	0.085625395	Insignificant
	Mm00485472_m1	Rac2	RAS-related C3 botulinum substrate 2	1.338	0.03895043	Flat
	Mm00450933_m1	Rala	v-ral simian leukemia viral oncogene homolog A (ras related)	1.035	0.8661943	Insignificant
	Mm00469677_m1	Ralb	v-ral simian leukemia viral oncogene homolog B (ras related)	1.339	0.17033182	Insignificant
	Mm00522941_m1	Rapgef3	Rap guanine nucleotide exchange factor (GEF) 3	-	-	N.A.
	Mm00834507_g1	Rhoa	ras homolog gene family, member A	1.066	0.6128333	Insignificant
*	Mm00782638_s1	Rplp2	ribosomal protein, large P2	1.775	0.006717769	Flat
	Mm00450234_m1	Scarb1	scavenger receptor class B, member 1	1.463	0.0030187257	Flat
	Mm00435860_m1	Serpine1	serine (or cysteine) peptidase inhibitor, clade E, member 1	0.763	0.364854	Insignificant
	Mm00486060_m1	Sftpd	surfactant associated protein D	1.061	0.8901841	Insignificant
	Mm00488332_m1	Siglec1	sialic acid binding Ig-like lectin 1, sialoadhesin	0.068	0.000068767535	Down-regulated
	Mm02525668_u1	Sirpb1a	signal-regulatory protein beta 1A	0.112	0.001369061	Down-regulated
	Mm00454684_m1	Stab2	stabilin 2	-	-	N.A.
	Mm00452814_m1	Stx18	syntaxin 18	0.943	0.5024671	Insignificant
	Mm01333032_m1	Syk	spleen tyrosine kinase	1.216	0.09423082	Insignificant
*	Mm00446973_m1	Tbp	TATA box binding protein	1.284	0.32984367	Insignificant
*	Mm00441941_m1	Tfrc	transferrin receptor	1.705	0.1138525	Insignificant
	Mm00436987_m1	Tgm2	transglutaminase 2, C polypeptide	0.556	0.4245617	Insignificant
	Mm00844508_s1	Ticam1	toll-like receptor adaptor molecule 1	1.168	0.45567507	Insignificant
	Mm01207404_m1	Tlr3	toll-like receptor 3	0.293	0.00022732517	Down-regulated
	Mm00446193_m1	Tlr9	toll-like receptor 9	0.622	0.05131324	Insignificant
	Mm00443258_m1	Tnf	tumor necrosis factor	0.448	0.025960745	Down-regulated
	Mm00441906_m1	Tnfsf11	tumor necrosis factor (ligand) superfamily, member 11	-	-	N.A.
	Mm00807071_m1	Vamp7	vesicle-associated membrane protein 7	1.085	0.91970754	Insignificant
	Mm01232047_m1	Vav1	vav 1 oncogene	1.100	0.44238567	Insignificant

	Mm00494167_m1	Was	Wiskott-Aldrich syndrome homolog (human)	1.065	0.76816595	Insignificant
	Mm00437347_m1	Wnt5a	wingless-type MMTV integration site family, member 5A	-	-	N.A.
